# Objective and subjective responses to motion sickness: the group and the individual

**DOI:** 10.1007/s00221-020-05986-6

**Published:** 2020-11-29

**Authors:** Tugrul Irmak, Daan M. Pool, Riender Happee

**Affiliations:** 1 Cognitive Robotics Department, Faculty of Mechanical, Maritime and Materials Engineering, Delft University of Technology, Delft, Leeghwaterstraat The Netherlands; 2 Control and Simulation Section, Faculty of Aerospace Engineering, Delft University of Technology, Delft, Kluyverweg The Netherlands

**Keywords:** Motion sickness, Repeatability, Modeling, Internal/external vision, Physiological measures

## Abstract

We investigated and modeled the temporal evolution of motion sickness in a highly dynamic sickening drive. Slalom maneuvers were performed in a passenger vehicle, resulting in lateral accelerations of 0.4 g at 0.2 Hz, to which participants were subjected as passengers for up to 30 min. Subjective motion sickness was recorded throughout the sickening drive using the MISC scale. In addition, physiological and postural responses were evaluated by recording head roll, galvanic skin response (GSR) and electrocardiography (ECG). Experiment 1 compared external vision (normal view through front and side car windows) to internal vision (obscured view through front and side windows). Experiment 2 tested hypersensitivity with a second exposure a few minutes after the first drive and tested repeatability of individuals’ sickness responses by measuring these two exposures three times in three successive sessions. An adapted form of Oman’s model of nausea was used to quantify sickness development, repeatability, and motion sickness hypersensitivity at an individual level. Internal vision was more sickening compared to external vision with a higher mean MISC (4.2 vs. 2.3), a higher MISC rate (0.59 vs. 0.10 min^−1^) and more dropouts (66% vs. 33%) for whom the experiment was terminated due to reaching a MISC level of 7 (moderate nausea). The adapted Oman model successfully captured the development of sickness, with a mean model error, including the decay during rest and hypersensitivity upon further exposure, of 11.3%. Importantly, we note that knowledge of an individuals’ previous motion sickness response to sickening stimuli increases individual modeling accuracy by a factor of 2 when compared to group-based modeling, indicating individual repeatability. Head roll did not vary significantly with motion sickness. ECG varied slightly with motion sickness and time. GSR clearly varied with motion sickness, where the tonic and phasic GSR increased 42.5% and 90%, respectively, above baseline at high MISC levels, but GSR also increased in time independent of motion sickness, accompanied with substantial scatter.

## Introduction

Motion sickness is a maladaptation syndrome where aggravating motions trigger autonomic symptoms such as salivation, dizziness, headaches, panting, hot/cold flushes, stomach awareness, nausea and vomiting. Chronic exposure to sickening motions may lead to the sopite syndrome, which is associated with lethargy, fatigue and drowsiness (Bertolini and Straumann 2016; Lackner 2014). Eliminating motion sickness, particularly in ships and trains, has been long sought after and automated vehicles are another mode of transport added to this list. The public outlook towards automated driving is positive, fueled by the foreseen freedom automated vehicles can provide. Users wish to be able to engage in activities that do not necessitate road observation. However, as shown in a multitude of previous studies (Turner and Griffin 1999; Kuiper et al. 2018; Salter et al. 2019), motion sickness becomes a major constraint when taking the eyes off the road. Fortunately, there are conceivable ways of reducing sickness incidence. For instance, route and path planning algorithms as well as smart active suspension controllers installed in future modes of transport may help ease symptoms. However, to be successful, these technologies rely on the accurate modeling of motion sickness, taking in to account the motion and the visual viewing conditions.

The modulating effect of viewing condition on sickness has been demonstrated. Griffin and Newman (2004), driving on Southampton urban roads, observed no significant differences between internal vision, which is when the passenger can only see inside the cabin, and blindfolded vision. However, both conditions showed approximately twice as high subjective sickness ratings as the external vision. Likewise, Butler and Griffin (2009) found internal/blindfolded approximately twice as sickening as external vision in combined fore-aft acceleration and pitch rotation at 0.1 Hz. However, Butler and Griffin (2006) found no differences in sickness between internal, external and blindfolded for pure fore-aft accelerations at 0.1 Hz 0.89 ms^−2^ rms. This suggests that the alleviating effect of external vision only occurs when the motions experienced lead to a perception of rotation. One of the aims of this study is, therefore, to quantify the influence of viewing condition on sickness during complex rotational and translational motions, as present during cornering.

The mathematical modeling of motion sickness thus far focused on population averaged measures of motion sickness. To name two, these may be in the form of motion sickness incidence (MSI) (O’Hanlon and McCauley 1974) and motion sickness rating (MSR) (Griffin and Howarth 2000). The computation of this averaged illness inherently transforms the data, such as to exhibit a converging sickness response profile, as seen in much of the literature (Bijveld et al. 2008; Cian et al. 2011; Bos et al. 2005). However, individuals show a range of responses that can broadly be categorized as convergent, i.e., to saturate at a certain level, or divergent, with a rapid increase towards emesis described by Bock and Oman (1982) to be an “avalanche” effect. After exposure to sickening motions, humans need minutes or even hours to recover. Within this recovery period, humans display “hypersensitivity” to new motion stimuli (Oman 1990). The modeling of individual dynamics is used widely in cybernetic research, one example being driver modeling (Barendswaard et al. 2017; Mars et al. 2011; Van Der El et al. 2017). This study aims to use a similar approach to motion sickness. The use of individual responses for modeling hinges on the assumption that individual responses are repeatable. That is, the response dynamics is a largely deterministic function of the motion stimuli, while the influence of internal psychological factors on the day-to-day response variation is much smaller than the inter-individual variation. The current study, therefore, aims to quantify the repeatability of motion sickness responses to sickening stimuli.

Posture is shown to be an important factor in sickness severity. Participants exposed to earth horizontal vibrations when upright reported sickness responses factor four greater than for lying supine (Golding et al. 1995). The dependency of sickness on posture is in concordance with the postural instability theory proposed by Riccio and Stoffregen (1991). They state that animals become sick in situations in which they do not possess adequate control strategies that are required for the maintenance of postural stability, and that postural instability precedes the symptoms of motion sickness, where postural instability is necessary to produce sickness symptoms. Following the postural instability theory, a supine posture is less sickening because it is an inherently more stable configuration. Several experimental studies support the postural instability theory using visual optic flow as method of inducing sickness (Stoffregen and Smart 1998; Villard et al. 2008; Smart et al. 2002; Stoffregen et al. 2014). Studies using inertial motions have not found significant postural differences between sick and well groups (Tal et al. 2010; Stoffregen et al. 2013; Varlet et al. 2015). However, these experiments were carried out under long duration low intensity sickness conditions on ships. To the authors knowledge, the effect of posture on sickness, or that of sickness on posture, has not been fully quantified under more repeatable and sickening conditions. Hence, we studied 3D posture maintenance of the head in our driving experiments.

Models of sickness have predominantly relied on subjective measures of illness or objective vomiting incidence. However, both have their misgivings. For instance, the former is affected by participant uncertainty on how they are feeling, which is an issue in particular at lower sickness levels. The latter, on the other hand, cannot yield any information on individual responses, nor the time history of sickness. Subjective ratings also have a low time and sickness resolution. For instance, the MISC rating scale (Bos et al. 2005) usually ranges from 0 to 7 from no discomfort to moderate nausea, and reliable estimates may only be given in $\approx$ 30-s intervals. Moreover, querying the MISC may even affect sickness development (due to increased introspection) and performance on other experimental tasks. Accurate modeling, however, ideally requires objective measures with high time and sickness resolution. Physiological measurements such as electrocardiography (ECG) using the low–high frequency ratio (LF/HF), of heart rate variability (HRV), heart rate (HR) and galvanic skin response (GSR) and also postural stability, may be appropriate for this purpose. Many studies have evaluated HR (Cowings and Toscano 1993; Holmes and Griffin 2001; Mullen et al. 1998), HRV (Holmes and Griffin 2001; Himi et al. 2004; Lin et al. 2011; Ohyama et al. 2007; Dahlman et al. 2009) and GSR (Wan and Hu 2003; Dahlman et al. 2009; Himi et al. 2004) as measures of motion sickness. Under the large range of motion sickness levels encountered by participants in this study, we aim to clarify how these physiological measures correspond to sickness.

Overall, this study aims to (1) quantify the differences for internal and external vision conditions during complex motion experienced in cornering, (2) quantify the repeatability of individual motion sickness responses in time including hypersensitivity, (3) validate Oman’s nausea model to describe the individual time evolution of sickness, and (4) relate objective physiological and kinematic variables such as HR, HRV, GSR, and postural stability to subjective sickness rating.

## Methods

The present study is comprised of two complementary experiments exposing participants to a sickening drive. Experiment 1 aims to quantify the effect of visual view on motion sickness. Experiment 2 aims to quantify individual response repeatability and hypersensitivity.

### Participants

In total, 24 participants took part in the first experiment, where 18 participants completed both internal and external visual conditions. Of the 24 who took part, 6 participants were female and 17 were male. Of the 18 participants completing both internal and external vision conditions, 4 were female 14 were male and. 3 of the 18 participants were experimenters themselves. These 18 participants had a median motion sickness susceptibility questionnaire (MSSQ) score of 16 indicating that they were of above average motion sickness susceptibility. The MSSQ was not used in the participant selection process.

For the second experiment, 17 participants took part, of whom 13 participants completed all 3 sessions investigating repeatability. Of these 13 participants, 3 were female and 10 were male. None of the participants were experimenters. The median MSSQ score of these 13 participants was 5 indicating they were of below average susceptibility.

No participants performed both experiments. All participants had normal or corrected to normal vision. None of the participants reported any vestibular disorders. The mean age for the two experiments was 26.1 years (STD = 8.2). Lastly, all participants were asked to refrain from recreational drug consumption, including alcohol and caffeine, from at least 24 h prior to the experiment.

All participants provided written informed consent prior to participation. The experimental protocols for both Experiments 1 and 2 were approved by the Human Research Ethics Committee of TU Delft under application numbers 420 and 521.

### Apparatus

**Fig. 1:**
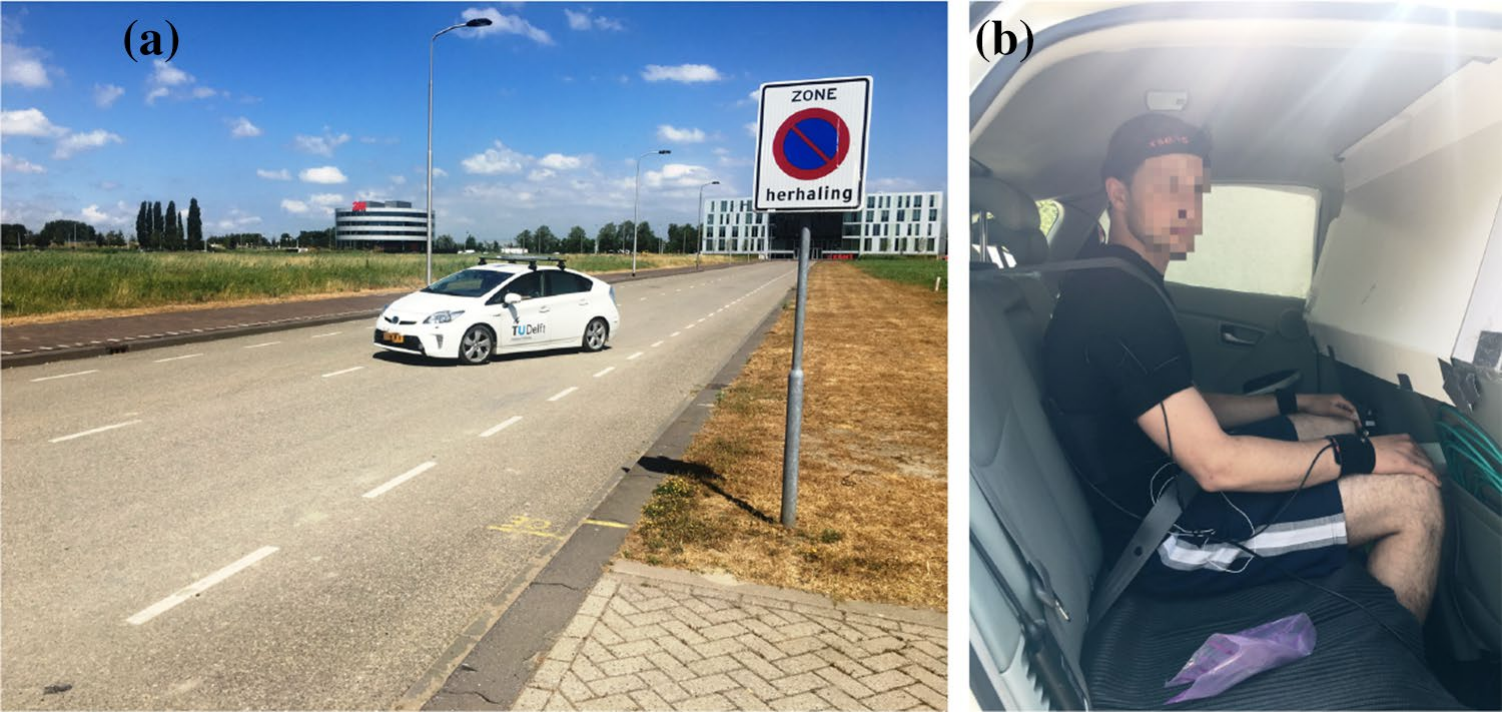
Experimental setup. Left is the Prius 2013 model on the test road used during the experiments in the process of performing a slalom. Right shows the internal vision condition where the external view to the sides and front is blocked

Participants were seated at the middle back seat of a Toyota Prius (2013 model), see Fig. 1, on top of a friction mat to prevent lateral slippage at the buttocks/seat interface. The vehicle was instrumented with a 6-DoF Inertial Measurement Unit (IMU) mounted at the bottom of the rear middle seat, below the seated participant, recording acceleration, orientation and angular velocity at a frequency of 100 Hz.

In the experiments described in this paper, we tested sickness development in two visual conditions, i.e., internal vision and external vision. In the external vision condition, participants had a normal view of the road ahead through the front and side windows of the car. In the internal vision condition, the front view was obscured by a cardboard panel, affixed to the front seats. In addition, the side view was obscured by cardboard templates stuck to the windows. This effectively obstructed all views of the road and the movement of the vehicle.

### Task and stimuli

For both experiments, the vehicle described in "Apparatus" was used to drive slalom trajectories. For this purpose, a closed road of length 240 m and width of 10 m was used. The slaloms were of an amplitude of 3.5 m and a frequency of 0.2 Hz. The longitudinal velocity of the vehicle was held approximately constant, via manual control, at 25 km/h. The slalom frequency was maintained with the help of a metronome. The road markings at the road boundary were used as a guide mark for the motion. As a consequence, the participants experienced lateral accelerations with a peak amplitude of 0.4 g. At the end of the available road, a 3-point turn was performed and the slalom restarted. Before each 3-point turn, 8 slaloms were performed. Such a single test stretch was completed in approximately 40 s. Each 3-point turn took 8–10 s to complete. The relatively large excitation used in both experiments aimed to obtain a robust sickness response and a large distribution of sickness ratings within the population. This supports the analysis and modeling of individual sickness development in time. In both experiments, participants were told to assume a relaxed posture with their feet placed wide apart, their hands on their knees, with a straight back looking directly out of the front wind screen for the external vision condition, or at the cardboard cover occluding the wind screen in the internal vision condition. The air condition regulating the internal temperature of the vehicle was set to $18^{\circ }$C.

In this paper, we define a “session” as an experimental block that starts from the time at which a participant comes in to the experiment staging room on a particular day, until they have completed the experiment and have been debriefed, leaving the staging room. An “exposure” refers to the motion exposure. In the Experiment 1, there is only one motion exposure. In the Experiment 2, there are two: the first motion exposure, followed by a rest, which is followed by a second motion exposure to test for hypersensitivity.

In Experiment 1, all participants were tested in the two different visual conditions (external and internal vision) with an average interval of 1 week between both sessions. In Experiment 1, the sickening drive lasted for a total of 30 min, or until the participants reached a MISC rating of 7 or otherwise asked to terminate the experiment. The order of testing for the two visual conditions was balanced between participants.

Experiment 2 consisted of three repeated identical sessions, where participants experienced the sickening drive in the internal vision condition only. A 1-week interval was planned between each session aiming to eliminate effects of habituation. Where Experiment 1 consisted of only a single (maximum 30-min) motion exposure within each session, in Experiment 2, a second exposure was included in each session to measure hypersensitivity. Each session of Experiment 2 started with the pre-experiment drive to the test road, followed by the first motion exposure (M1). After a rest period (R1), which brought the participants back down to a MISC rating of 2, they were given the option to proceed with the second motion exposure. This second motion exposure (M2) was terminated after 15 min or when participants reached severe motion sickness (MISC of 7) or otherwise requested for the termination of the experiment. After the second exposure, a second rest period (R2) was followed by the post-experiment drive to the staging room.

In both experiments, participants were tasked with reporting their sickness level. For this purpose, the MISC scale (Bos et al. 2005) was used as a subjective indicator of motion sickness during the experiment. The MISC scale is a commonly used symptom based rating system and provides a measure of sickness which is comparable between participants. The participants were first familiarized with the MISC rating at the briefing room, at the start of each session. In Experiment 1 the participants were asked their MISC rating in 1 min intervals, during and after the sickening drive. The participants’ verbal answers, consisting of a single integer MISC rating, were recorded with pen and paper . In Experiment 2, the interval for MISC requests was brought down to 40 s. An automated system provided an auditory cue asking “MISC?” and recorded the participants’ verbal answers. The recordings were then manually processed, which proved to be more robust than the verbal approach in Experiment 1. In Experiment 2, the MISC was asked from the moment the participant entered the vehicle to the moment they returned to the staging ground for debriefing.

### Instrumentation for physiological measurements

The kinematics of the participants were measured via the use of the Xsens full body inertial motion capture suit (Xsens 2020), recording at 240 Hz. The motion capture suit consists of 21 inertial measurement units distributed across relevant body segments. The raw sensor recordings are paired with a bio-mechanical model of the human skeleton. This model is calibrated to the participant upon initialisation. The calibration procedure consists of taking a neutral, N-pose with arms by the side with a straight upright posture for a few seconds before walking for a few paces with a natural gait before turning and returning back to assuming the N-pose at the point walking started. The motion capture system processes the raw sensor readings via the use of this biomechanical model before returning joint angular positions and earth referenced orientations, accelerations and angular velocities.

The ECG and the GSR measurements were both sampled at a frequency of 1 kHz via a TMSI Mobita amplifier. The ECG was recorded via 3 leads at the V1, V2 and V3 locations (Rosen et al. 2014). The ground was attached to the participant wrist with a wet electrode wrist band. The GSR was recorded via 2 gel electrodes at the index and the middle fingers.

### Data pre-processing

Head roll was previously reported to relate to motion sickness (Wada et al. 2018). As the lateral vehicle accelerations in our experiments primarily elicit head roll, measured head roll was used as the main indicator of postural instability. We evaluated head roll around the slalom frequency using a 0.15–0.22 Hz band-pass filter. To compensate for variation in vehicle motion we scaled head roll to the vehicle lateral acceleration. The scaling was done by dividing the moving-average (60-s window) root mean square of head roll, with the moving-average root mean square of the vehicle lateral acceleration. Due to the scaling by the rms of the vehicle acceleration at zero acceleration periods, the relative head roll may be very high. These outliers caused by the scaling were removed.

ECG gives information on HR and HRV which may be of use in detecting the development of motion sickness. For this purpose, raw ECG was first recorded and then detrended by fitting a 6th-order polynomial. The subsequent trace was then transformed using a sym4 wavelet. The 4th and 5th length scales were taken and an inverse maximal overlap discrete wavelet transform was performed. The resulting output was squared and fed in to a peak detection algorithm. The detected peaks were manually checked and any false positive and false negatives were manually corrected. The HR and HRV were calculated. The latter is the time difference in seconds between adjacent R–R peaks. It was then interpolated using the 5th-order Lagrange interpolation with a re-sampling frequency of 10 Hz. The heart rate was then filtered with a band-pass filter of pass-band frequency of 0.01 Hz and stop-band frequency of 0.02 Hz. The instantaneous LF/HF was calculated by first computing the Choi–Williams distribution of the HRV, and then band-pass filtered with a pass-band frequency of 0.01 Hz and stop-band frequency of 0.02 Hz.

The measurements from the GSR device, which are first given in micro-volts, were first converted to conductance measured in microsiemens ($\upmu$S). The raw GSR files were processed using the batch processing command in Ledalab (Benedek and Kaernbach 2010a, b). Ledalab is an open source source MATLAB toolbox. It decomposes the GSR signal in to its tonic (low frequency) and phasic (high frequency) components, respectively (Benedek and Kaernbach 2010b). To quantify the strength of the phasic GSR its absolute value was integrated over the time span between the MISC prompts by deconvolving the original GSR signal.

**Fig. 2:**
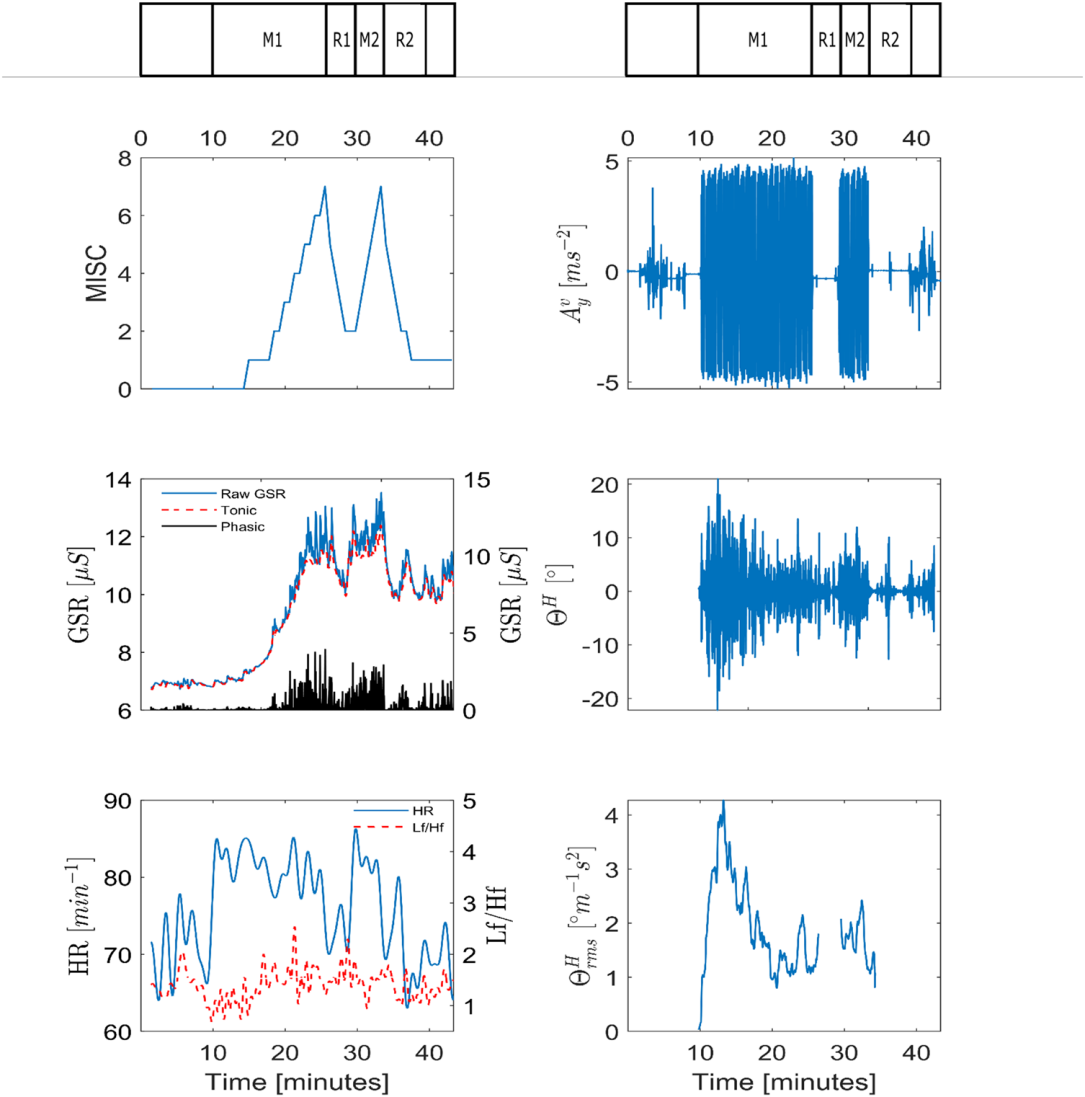
Example response of participant 11 for session 3 in Experiment 2. MISC level and vehicle acceleration are shown in the top row. The middle row shows the raw, tonic and phasic GSR, as well as the head roll. Likewise, the bottom row, shows the heart rate, LF/HF ratio as well as the ratio of the head roll rms and vehicle lateral acceleration rms

Figure 2 shows example measurements of the MISC, vehicle lateral acceleration, and physiological data from Experiment 2 for participant 11 in session 3. In this paper, the signals shown for the phasic and the tonic GSR, as well as heart rate and LF/HF ratio of heart rate variability and head roll rms ratio, are analyzed to see if they are predictive of the MISC level.

### Data analysis

#### Mean MISC and MISC rate

To answer our research questions on the effects of visual viewing condition, repeatability of sickness response, and hypersensitivity, the severity of sickness must be quantified. While many different metrics have been used for this, in this study, we use the mean MISC and the MISC rate as parameters that quantify sickness.

The mean MISC is calculated by averaging all MISC ratings over the intended M1 exposure period. If a given participant finished a motion exposure prematurely (by reaching a MISC rating of 7), then for the remainder of the 30-min duration, a value of 7 is used for this participant in the calculation of the mean MISC. This padding technique has been employed in a number of earlier sickness studies (Webb and Griffin 2003; Griffin and Newman 2004). Figure 4a shows the dropout rate in Experiment 1. Figure 4c, shows that averaging using a MISC of 7 for missing data creates a MISC as function of time that deviates from individual curves in Fig. 4d. However, the alternative of omitting missing data, indicated with dashed lines in Fig. 4c, is even less appropriate and results in a reduction in mean sickness. Hence, we use the mean MISC as a robust measure per exposure and use MISC rate and Oman’s model to assess MISC development in time for each individual exposure.

The MISC rate is calculated as a simple linear measure of how quickly the MISC has increased. It is calculated from the difference in MISC scores between two relevant time points divided by the time interval between these two time points in minutes. Here, we report MISC rate from the start towards the end of motion exposures M1 and M2. Both the mean MISC and MISC rate are effective metrics for describing group differences in sickness response.

#### Oman’s Model

Literature shows various ways of modeling the time response of motion sickness. In all cases a sensory conflict term (Oman and Cullen 2014) is integrated over time (Dai et al. 2010; Bos and Bles 1998). For the analysis of the repeatability of sickness responses in our Experiment 2, we took the rectified lateral vehicle acceleration as the sensory conflict input. This is appropriate as no visual view of the car motion was available (so all sensed accelerations were conflicting) and because the sickness susceptibility for lateral perturbations shows a plateau from 0.03 to 0.3 Hz (Donohew and Griffin 2004). The bandwidth of our excitation was within this range, centered narrowly around 0.2 Hz, and as the lateral acceleration was the dominant motion, this was deemed a good proxy for internal sensory conflict. Even if the conflict were some scaled factor of the vehicle acceleration, model tuning would scale this appropriately to the output MISC rating. The integration of this conflict term may take the form of a simple integrator as in Dai et al. (2010), or a second-order system as in Bos and Bles (1998). However, these are simple mechanisms which do not explain the observations of hypersensitivity that occurred during this study. Hypersensitivity as a phenomena was first investigated by Oman (1990) who developed a model of motion sickness that aimed to account for both the initial rise in sickness and the hypersensitivity that occurred after re-exposure to sickening stimuli.

**Fig. 3:**
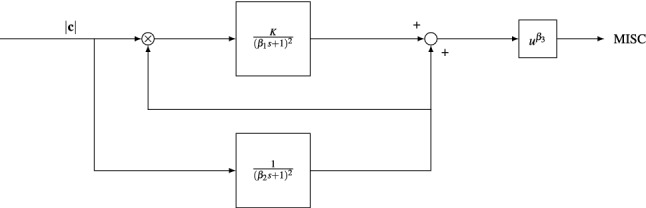
Adapted Oman’s model of motion sickness development in time. The rectified conflict signal |*c*| is fed in to the model. There is a fast (upper) path and a slow (lower) path. The slow path multiplies with the conflict as the gain of the fast path. Both systems are 2nd order with repeated poles. The fast and slow path are then summed and the result is raised to a power where $\beta _3 >0$

The model as documented by Oman (1990) is parameterized to have $\beta _1 = 60$, $\beta _2 = 600$, $\beta _3 = 2$. However, these parameters had not been validated in a structured manner, and in Oman et al. (1986), a potential value for the gain *K* was not identified. We, therefore, leave them as free parameters that are tuned to MISC responses for each individual and session of experiment 2. However, before doing so, we made minor adjustments to the model (Fig. 3). We set the numerator of the fast path to 1 and removed the constant gain of 5 from the slow path. This allows us to compute a unique gain for *K*. We have also set the power term $\beta _3$ to 1. This is because, when fitting the original model, we found the $\beta _3$ parameter to be redundant for the MISC scale we used. Upon inspecting the results, we simplified this model further. There was a strong correlation ($\rho = 0.69$) between the time constants of the slow and fast paths, where $\beta _2 = 7 \beta _1$. We, therefore, used this substitution to simplify the original four-parameter model into the two-parameter one shown in Fig. 3. The substitution greatly simplified the model, while inflating the model fit error by only 1%.

#### Kinematic and physiological variables

In this paper, we aim to find the relationship between sickness, given by the MISC rating, and head roll, heart rate, LF/HF ratio of heart rate variability, and GSR. To obtain a larger dataset, experiments one and two are combined. Only the first motion exposure of each session is utilized and, because of a potential confound with hypersensitivity and time related effects, M2 in Experiment 2 is not used. Although there are two different visual conditions in the first experiment, we show that this makes no significant difference to the head roll response. For the physiological measurements, this again is of no importance as we assume that vision does not directly influence these physiological measures.

For the analysis, the MISC rating at a given time is paired with the associated physiological and kinematic variable for the point in time the MISC was recorded. This allows us to perform a regression analysis on these variables and the MISC. As there were some dropouts for whom the experiment was prematurely terminated due to reaching a MISC level of 7 (moderate nausea), and missing or partially missing physiological data due to technical difficulties, all available data were aggregated to paired MISC and physiological responses.

We then built a linear mixed model with random intercepts that relates the chosen kinematic/physiological variable with the associated MISC value. It is seen from the raw data that time since the start of the experiment may also have an influence on these variables. For instance, heart rate is influenced by arousal, which is particularly high at the start of the experiment, likewise, the tonic GSR may increase as the participant sweats due to the exertion required to stabilize posture. To control for this, the effect of time is also modeled. To make sure the regression coefficients are not dependent on each other we performed a multicollinearity test before each regression. Indeed, most responses are similar to those seen in Fig. 2 and are well approximated with a linear model. As the regression residuals have a fat tail and are not normally distributed, we bootstrap the regression, using 4000 iterations of random-x resampling in MATLAB (Fox 2002). This gives the 2.5% and 97.5% percentile confidence intervals for the regression coefficients.

## Results

### Experiment 1: internal–external vision group response

The motion sickness incidence in horizontal motion plateaus between the frequencies 0.03 and 0.3 Hz (Donohew and Griffin 2004). A two-tailed paired *t* test was conducted on the average lateral vehicle acceleration power over this frequency range between the conditions in Experiment 1.

**Fig. 4:**
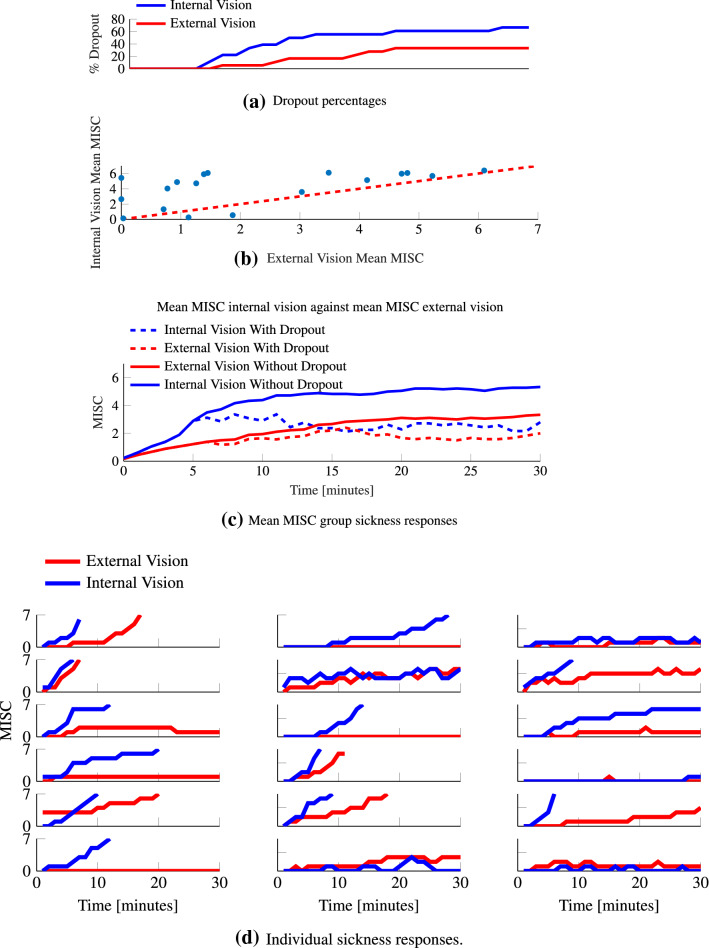
MISC responses for both internal and external vision conditions recorded during the sickening drive in Experiment 1. **a** Shows the dropout % as a function of time. **b** Shows mean MISC for internal vision against external vision. **c** Shows the group-average mean MISC response calculated by omitting the dropouts (dashed lines) and replacing dropouts by MISC 7 (solid lines). **d** Shows the individual responses

There was no statistically significant difference between the visual conditions (*t* = $-0.347$, *p* = 0.733, *df* = 17). As the applied vehicle motion stimuli are similar, human responses can be compared between the visual conditions. Figure 4a shows the dropout rate for the two visual conditions. For internal vision this was 66% and for external vision this was 33%. Figure 4c shows the mean MISC for internal and external vision conditions. As can be verified from Fig. 4d, a large number of participants reached a MISC of 7 within 30-min. As explained in "Mean MISC and MISC rate", for these participants a constant MISC of 7 was taken to compute the mean group MISC up to 30 min. Figure 4b shows substantial scatter when relating the mean MISC for internal and external vision. Apparently, several participants develop noticeable sickness with internal vision only; while for others, the sickness with internal vision is close to the level reached with external vision.

Effects of visual condition were evaluated at each sample time using a paired Wilcoxon signed-rank test since MISC is not normally distributed. The sickness responses for internal and external vision diverge significantly at the 5th minute (*p* = 0.0166, SR = 12, *n* = 18). At the end of motion exposure, the mean MISC for internal vision is 5.3 (STD = 2.51) and for external vision this is 3.3 (STD = 2.64). The mean MISC across time is 4.2 (STD = 1.5) for internal vision and 2.3 (STD = 0.95) for external vision. Therefore, throughout the duration of this experiment, internal vision is significantly more sickening than external vision. Due to capping MISC at 7 for those participants who could not complete the 30 min exposure, the difference is in fact greater than shown here. MISC rate provides a second measure that is not distorted due to the capping of MISC at 7. The median MISC rate for internal vision is 0.59, and for external vision it is 0.1. This is a large and significant (Wilcoxon signed-rank test *p* = 0.011) difference. The mean MISC, MISC rate and the dropout rate indicate that, compared to the external vision condition, sickness develops faster and to a greater level in the internal vision condition. However, the effect of visual condition varies strongly across participants.

Figure 4d shows the MISC responses of individual participants for the two visual conditions. There is a great variety of responses which, by the averaging in Fig. 4c, is lost. From visual inspection, the responses seem to follow two categories. One is an exponentially divergent, “hockey stick”-type response indicative of an unstable sickness response. The other is an exponentially convergent type response indicative of a stable sickness response. To show this, we fit a function of the form $at^b$ to these responses. For $b \ge 1$ the response is divergent and for $b<1$ the response is convergent. The *b* parameter is distributed over a large range (0–6.2). Moreover, for some participants, due to the coarseness of the subjective ratings in both the temporal and sickness resolution, the parameters cannot be estimated accurately. In addition to this, *b* cannot be estimated for the participants that do not get sick. For these participants we set *a* and *b* to zero. This makes clustering individuals difficult. However, it is likely that *b* is a parameter that depends on the magnitude of the sensory conflict. Supporting this, using a paired Wilcoxon signed-rank test, we see that the median *b* for internal vision (higher sensory conflict) is 0.93 which is significantly greater $p= 0.0475$ than the 0.39 in external vision (lower sensory conflict). Indeed, the means 1.46 and 0.69, respectively, indicate complex amplitude dependent dynamics for sickness progression. Capturing such richness in response requires models whose parameters can be tuned to fit individual responses.

### Experiment 2: model fit

We first validate the adapted Oman model shown in Fig. 3 by fitting it to the responses observed from Experiment 2. The fitting was done to the individual session responses of 13 participants. This is because participants 7, 16 and 17 dropped out of session two and/or session three and the vehicle IMU recording of participant 10 was incomplete. To evaluate the model fit, we used the Symmetric Mean Absolute Percentage Error (SMAPE):

$$\begin{aligned} \text {SMAPE} = 100\frac{\varSigma ^n_{t=1}|F_t - A_t|}{\varSigma ^n_{t=1}|A_t + F_t|}, \end{aligned}$$

where $F_t$ and $A_t$ are the fitted and the actual value at time *t*, respectively. This error metric is well protected against outliers and treats both over- and underestimation in an unbiased manner.

The model fit has an average SMAPE of 11.4%. This means that while the model captures most variations, there are certain dynamics that it does not. Looking at the errors per period, the SMAPE for M1, R1, M2 and R2 is 12.3%, 8.8%, 8.3% and 23.4%, respectively. Applying multiple Mann–Whitney *U* tests between R2 and R1 errors, the *p*-values are calculated to be $p < 0.005$. Likewise, between M1 and M2, $p =0.003$. With a Bonferroni correction, the critical value is $\alpha =0.025$ confirming significance of both effects. The model has more difficulty capturing the MISC change during R2 compared to R1. This may be because R2 has inherently different dynamics than R1, or more plausibly, because the observation window is larger for R2, which causes the inaccuracy in the modeling of the rest period to become more apparent. For M1, the error is significantly larger than for M2, which could be due to the fact that M2 on average lasts a shorter amount of time.

### Experiment 2: repeatability of individual responses

For motion sickness to be modeled by dynamical equations, repeatability in response is important. Qualitatively, we can see from Fig. 6 that individuals have a high degree of repeatability between the consecutive sessions.

**Fig. 5:**
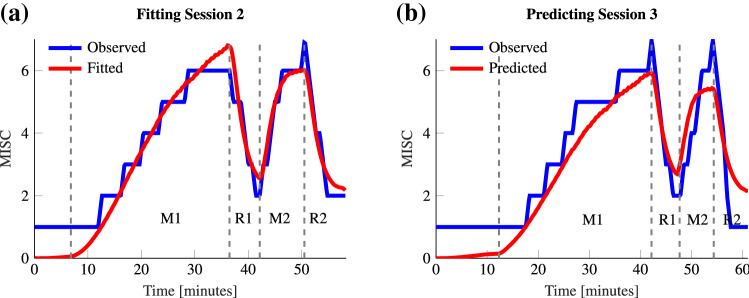
Example Oman model fit and predictions for the sickening drive data of Participant 6 from Experiment 2. Left shows the fitted model to the observed MISC of session 2 (SMAPE of 6.4%). Right shows the predicted MISC when the model parameters found for session 2 are used to predict the response of session 3 (SMAPE 11.7%)

This individual repeatability can be quantified by using Oman’s model (Fig. 3). To do this, the model was tuned to fit the sickness response of session 2 in Experiment 2. We verified how well, using accelerations from session 3 as input, the parameters of session 2 could predict the MISC ratings seen for session 3 (here session 2 and 3 were compared to reduce skewing effects of habituation that might be present during session 1 and session 2). An example is shown in Fig. 5. The SMAPE over the entire duration of the experiment averaged over 13 participants is 23.2% for session 3 using the parameters obtained by fitting the MISC for session 2. This shows responses to be repeatable over consecutive sessions. As a measure of how much more information individual responses gives us, this SMAPE can be compared to the average SMAPE when parameters obtained by tuning for session 2 of a participant is used to predict session 3 of any other participant. In this case the mean SMAPE is 46.6%. This clearly confirms the reduced accuracy of group-based models of sickness: individualized models of motion sickness can reduce the prediction error by a factor of 2.

### Experiment 2: hypersensitivity

**Fig. 6:**
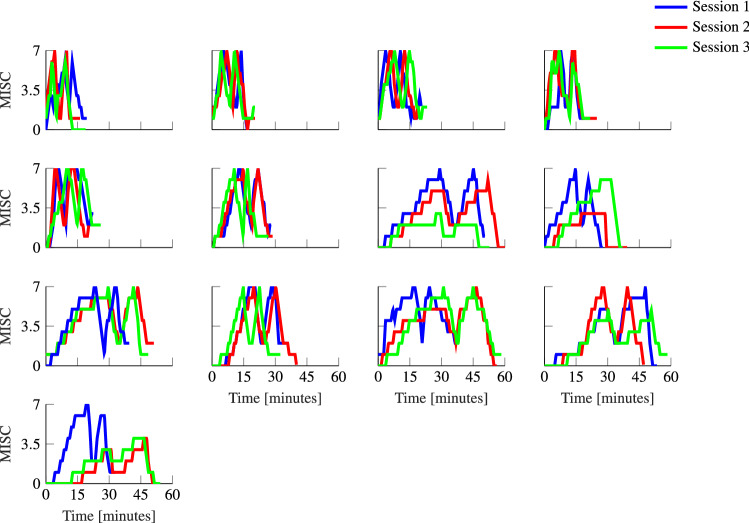
Individual responses to the sickening drive across three repeated sessions in Experiment 2

Hypersensitivity is seen to occur when after a brief rest participants who are exposed to further sickening motions respond in a much faster manner than during their initial exposure. This can clearly be observed in Fig. 6, with a much quicker rise in M2 than in M1. The median MISC rate was 1.02 s^−1^ for M2 and 0.395s^−1^ for M1 and differed significantly (*p* = 0.0421, Wilcoxon test). This indicates a much higher sensitivity in the second exposure.

The adapted Oman model was used successfully to model both the initial response (M1) and hypersensitivity (M2), with one set of parameters. Group average parameters are 441 and 2.18 for $\beta _2$ and *K*, respectively. The parameter $\beta _2$ corresponds to the decay time constant of hypersensitivity. This is, an average value of 441 s, or 7.35 min and Oman (1990) who reported time constants of approximately 14 and 10 min, respectively.

### Experiment 2: influence of parameters

**Fig. 7:**
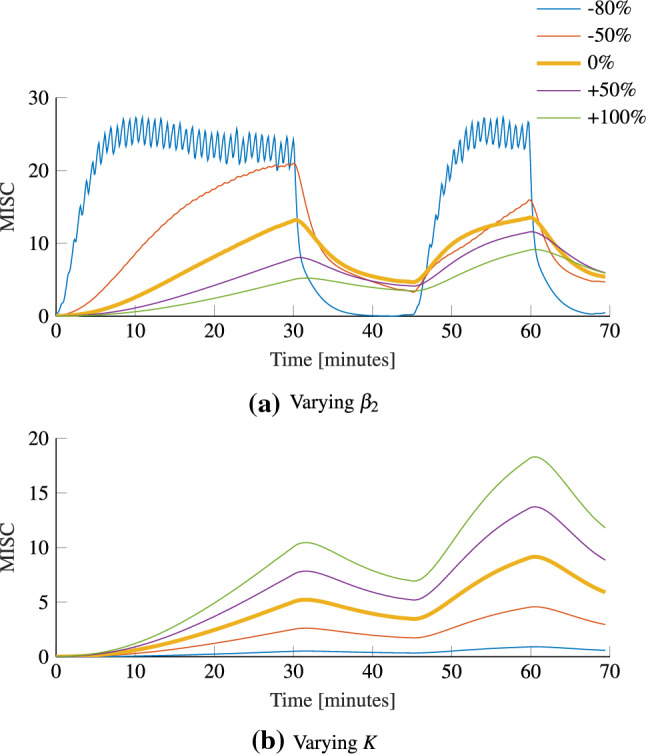
Effect of varying the group averaged parameters on the average sickness response; top shows the effect of varying the slow path time constant $\beta _2$. Bottom shows the effect of varying the gain *K*

To further support the sickness response modeling results, Fig. 7 shows the effects of varying the two parameters of the model ($\beta _2$ and *K*) with respect to their group average values on the model’s predicted sickness response. The top figure shows that lowering the time constant $\beta _2$ leads to a faster sickness development. As, for this paper, the slow path and fast path time constants are coupled by $\beta _2 = 7\beta _1$, reducing the value of $\beta _2$ reduces the value of $\beta _1$. Doing so reduces the damping of the sickness response. The lowest $\beta _2$ and $\beta _1$ show MISC fluctuations caused by the stop and turn performed after the slalom seen in Fig. 7. Varying *K* on the other hand, has no influence on the time response and *K* only acts as the gain on the amplitude of the sickness response. To conclude, the adapted version of the Oman model is seen to successfully characterize the full course of individual sickness response including hypersensitivity and its two parameters cause simple, interpretable changes in the modeled sickness response.

### Experiment 1 and Experiment 2: kinematic and physiological variables

#### Galvanic skin response

Motion sickness can cause an increase in both the tonic and phasic GSR, indicative of the fight or flight response (Mackersie and Calderon-moultrie 2016). In motion sickness experiments, both components may also be a function of time, in addition to sickness. To verify these effects, we use a mixed linear model of the form:

1 $$\begin{aligned} \mu S_{\text {tonic}}= \,& {} \alpha _0 + \alpha _1 {\text {MISC}} + \alpha _2 t + u_j + \epsilon _t, \end{aligned}$$

2 $$\begin{aligned} \mu S_{\text {phasic}}= \,& {} \gamma _0 + \gamma _1 {\text {MISC}} + \gamma _2 t + v_j + w_t, \end{aligned}$$

where $u_j$ and $v_j$ denote the random intercept for participant *j*, $\alpha _0$ and $\gamma _0$ are the intercepts, *t* is time and $\alpha _1$/$\alpha _2$ and $\gamma _1$/$\gamma _2$ are coefficients. The variance inflation factor between MISC and time is found to be 1.46 which is smaller than the value of 4 taken as the point where collinearity starts to become a concern. After fitting the linear mixed model, it was seen that the model residuals are not normally distributed, which reduces confidence in the statistics of the fit. Therefore, the model was bootstrapped using 4000 iterations of random-x resampling to derive the 2.5% and 97.5% percentile confidence intervals for the parameters.

For the tonic GSR, the effect of MISC is significant, with a mean coefficient 0.485 (CI between 0.379 and 0.586). This means, for an increase of MISC from 0 to 7, a mean rise of the tonic GSR by 3.4 $\upmu$S over the mean baseline of 8 $\upmu$S, which is an increase of 42.5%. The time coefficient is also significant with a mean value of 0.0006 $\mu$Ss^−1^ (CI between 0.0002 and 0.0010). However, its effect is smaller, resulting in an increase of the tonic GSR by 1.08 $\upmu$S over the baseline of 8 $\upmu$S (only 7%).

For the phasic GSR, the effect of MISC is also significant. The mean coefficient is 1.69 $\upmu$Ss (CI between 1.28 and 2.1). This means, for an increase of 11.83 $\upmu$Ss over the baseline of 13.2 $\upmu$Ss, an increase of 90%. The time coefficient is not significant, as the CI for $\gamma _2$ (between $-0.0019$ and 0.0005) crosses zero.

#### Heart rate and heart rate variability

For the analysis of HR and LF/HF ratio of HRV, we also follow the methodology explained in "Galvanic skin response". The variance inflation factor between MISC and time is found to be 1.80, i.e., smaller than the value of 4 taken as the point where collinearity starts to become a concern.

For HR, the effect of MISC is significant, with a mean coefficient of 0.466 (CI between 0.247 and 0.673). This means that for an increase of MISC from 0 to 7, there is a mean rise of HR by 3.3 bpm over the baseline of 87.4 bpm (only 3%). The time coefficient is also significant with a mean of $-0.0063$ (CI between $-0.0070$ and $-0.0055$). This means a reduction of 11.3 bpm (a drop of 12.9%) throughout the 30-min motion exposure. This drop in time due to relaxation of the participants masks the slight increase due to increased sickness.

For the LF/HF ratio the effect of MISC is also found to be significant. The mean coefficient is $-0.0463$ (CI between $-0.0279$ and $-0.0647$) which is a decrease of 18.52% over the baseline of 1.75 going from MISC 0 to 7. This is contrary to what would be expected based on the traditional view of LF/HF ratio where a decrease is indicative of a more relaxed state. The time coefficient is also significant with a mean of 0.00003 (CI between 0.00036 and 0.00022), a 3.1% increase over baseline. The time effect and the sickness effect are in opposite directions and so the net change in the LF/HF ratio is small.

#### Head roll

Head motion is taken as indicative of postural stability, which may relate to the development of motion sickness over time. Availability of visual cues may help in maintaining head stability and so have an effect on sickness development. The median head roll rms ratio for internal and external vision responses, across 11 participants from Experiment 1 for who valid head roll data was available, were found to be 2.05 and 1.60, respectively. However, this within-participant difference was not significant (*p* = 0.101, Wilcoxon test).

With no significant difference between internal and external vision conditions, for the remaining analysis, data from Experiments 1 and 2 are combined for all the participants, leading to data from 33 participants in total.

Within the experiment the MISC level is correlated with time. However, the variance inflation factor between MISC and time is found to be 1.09, i.e., well below the collinearity limit of 4.

For head roll there is a moderate effect of MISC, with a coefficient of 0.0212 (CI between $-0.011$ and 0.052). However this is not significant. Over a 7-point MISC scale, this is a mean increase of head roll rms ratio of 0.15 over the mean head roll rms at the intercept, giving a total head roll rms ratio of 1.85. For a peak lateral vehicle acceleration of 4 ms^−2^, this means an increase of head roll amplitude from 9.6$^\circ$ to 10.45$^\circ$, an increase of 8.9%. There is, however, a significant effect of time. Here, the mean coefficient is 0.000469 s^−1^ (CI between 0.000577 and 0.000365). Over the course of a 30-min experiment, starting at a baseline head roll of 9.6$^\circ$, due to the 4 ms^−2^ peak lateral vehicle acceleration, the mean increase in rms head roll is 14.4$^\circ$, a 50% increase. From this analysis, it can be concluded that for our data, the effect of time on the loss of head stability is stronger than the effect of MISC.

## Discussion

In this paper, we took to analyze motion sickness development in the time domain in contrast to the more widely used time averaged/instanced metrics in the literature. The richness of information has, therefore, allowed for greater insights in to the phenomenology of motion sickness.

### Internal–external vision

Internal vision was more sickening with a higher mean MISC (4.2 vs 2.3) compared to external vision, a higher MISC rate (0.59 vs 0.10) and more dropouts (66% vs 33%). Due to the non-linear relationship between MISC and comfort, however, this difference is likely much greater on a subjective comfort scale (such as the fast motion sickness scale Keshavarz and Hecht (2011)). Moreover, due to the mean MISC calculation, which capped those participants who had to prematurely end the experiment at a MISC of 7, the true difference is likely larger than found in this study. These findings concur with Griffin and Newman (2004), who found a similarly large difference in sickness rating between internal and external vision in natural driving conditions. The effects of visual condition can be due to both an increase in sensory conflict, caused by the removal of external world view, and the removal of anticipatory information regarding the vehicle trajectory.

The vehicle rotation with respect to earth in both roll and pitch was found to be negligible. Therefore, internal vision provides virtually identical visual cues for orientation with respect to gravity as external vision. The main difference between the information provided by internal and external vision lies in the visual translational and yaw information received. However, Bos et al. (2008) found in simple motion experiments that the absence of translational or yaw visual information does not impact sickness.

On the other hand, several studies show that the presence of anticipatory cues leads to a substantial reduction of motion sickness. Feenstra et al. (2011) found the mean MISC at the end of motion reduced from 2 to 0.5 when anticipatory information was added to a turbulent flight simulation. Likewise, Kuiper and Bos (2019) found a higher end of motion mean MISC of 3.6 compared to 2.3 when comparing unpredictable motion cues over predictable motion cues in a vestibular only sled experiment. Lastly, Karjanto et al. (2018) observed a major reduction from 10.4 to 1.4 on the motion sickness assessment questionnaire (MSAQ) when anticipatory information was provided to passengers undertaking left and right turns in the absence of external vision, compared to when such cues were not provided. Therefore, even though a number of studies agree on the alleviating effect of anticipatory cues on motion sickness, they do not arrive at the same effect size. The large effect sizes found by Karjanto et al. (2018) or Feenstra et al. (2011) may be due to differences in experimental stimuli or the scale used to measure motion sickness. Some scales such as MSAQ may have larger sensitivity within lower sickness levels than other scales such as the MISC. Therefore, an important consideration is the intensity of the sickening stimuli where Kuiper and Bos (2019) obtained MISC ratings between 2 and 3.5 whereas Feenstra et al. (2011) obtained MISC ratings between 0.5 and 2. The inhibitory effects of mechanisms such as anticipation may not scale proportionally with motion sickness severity or the magnitude of the sickening stimuli.

The mean MISC level is widely used and is useful in showing clear group responses, but it masks important individual dynamics. Some participants have a convergent MISC response, where the final MISC level seems to exponentially reach a terminal value, where others have a divergent, exponential response (Fig. 4d). The response dynamics may even be a function of the visual condition, where for instance, participant 16 has no response for external vision, but an exponential response for internal vision. Likewise, Participant 6 has an exponential response for internal and a convergent response for external. As previously mentioned, current models of motion sickness do not account for this complexity. Instead, they aim to predict the development of sickness at a population level. For instance, Bos and Bles (1998) predict the motion sickness incidence (MSI), defined as the percentage of the population that has vomited. However, we argue that as models of motion sickness are based on hypothesized neurological mechanisms, their predictions must be individual specific and related to the physiological symptoms individuals experience.

### Repeatability and Oman’s Model

It is often not feasible to build human models that are capable of individual forecasts. This is due to the number of internal and external factors that may yield to large response variability. Therefore, statistical models based on group metrics are commonly favored over dynamical system models. However, our results comparing repeated exposures over consecutive sessions show that the time response of motion sickness is largely repeatable. Knowing the response of a participant in the previous sessions allows for the prediction in the next session with a SMAPE of 23.2% for all four stages of the response combined during M1, R1, M2 and R2. If instead, a group average model is used, the average error fitting individual responses is 46.6% which is a factor of 2 greater than the error of the individual based models. This means motion sickness can be modeled on an individual level, where the more historic data available for that individual, the more reliable the model is.

For this repeatability analysis, we used an adapted version of the model of nausea developed by Oman (1990). We performed the first extensive validation of Oman’s model to predict the time course of motion sickness on the MISC scale, including hypersensitivity and rest phases of the exposure. We note that for hypersensitivity, the sickness rises consistently faster than during the first motion exposure. We estimate the time constant for hypersensitivity, $\beta _2$, to be 7.35 min. This is similar to the value of 10 min reported by Oman (1990). Golding and Stottt (1997) also measured an “objective” recovery time constant by measuring the loss in tolerable number of head movements during motion re-challenge. They found a decay time constant of 15 min. They report that, the decay in sensitivity is not monotonic and that there was a rise 2 h after stimulation. This rise is not predicted by Oman’s model. Indeed the presence of complex slow dynamics matches with our own informal observations. One participant in Experiment 1 withdrew from the study due to sickness persisting after many hours. Likewise, two participants in Experiment 2 reported resurgence of nausea in the following hours after the experiment. These were self-reports made by the participants and without the request of the experimenter, so the real number of those effected by the after motion dynamics, which seem to last in the order of hours to even days may be higher. This is, therefore, an important topic of future study.

We found that the fast path time constant was a factor 7 smaller than the slow path time constant. Reducing the value of the slow path time constant, therefore, caused both a reduction in the time to convergence of the sickness response and a reduction in the damping of the system (due to coupling with the fast path time constant). As can be seen from the negative placement of the system poles, the Oman model is stable and convergent. However, this does not stop it from providing good fits to divergent responses up to termination of the experiment. It is important to note that the gain of the fast path is a multiple of the input conflict; the subsequent amplitude of the sickness response is, therefore, a quadratic function of the conflict signal. This has important implications for mixed acceleration environments, such as traffic, where there may be an under- or overestimation of the effect of certain acceleration on the sickness response. Therefore, the relationship between stimulus intensity and individual sickness response dynamics should be investigated further. It may indeed be that there is a bifurcation in the nature of responses depending on the strength of the sensory conflict, which may also relate to the fact that some of our participants showed divergent responses for internal and convergent responses for external vision.

In the current study, we have observed that low time and sickness resolution impedes accurate model identification. That is, given the sparsity of data, multiple model fits may provide equivalent solutions. This was resolved by simplifying the model ($\beta _2 = 7\beta _1$ and $\beta _3=1$). This may compromise model validity in other conditions. For instance, the thresholding included in the original Oman’s model (excluded in this study) is indicative of the “functional vestibular reserve” (Graybiel 1969). This may be of particular importance when considering habituation or when modeling the effect of low amplitude motions that generate sub-threshold sensory conflicts. Future studies should keep the specificity of the current simplified model in mind.

To enhance the resolution in recording motion sickness, verbalized subjective ratings may be collected with a greater sampling frequency. Following an approach similar to Cleij et al. (2019), sickness ratings may be acquired in a continuous manner via the use of a dial. This approach may increase rating variability, but combined with verbalized ratings as an anchor, this can result in a valuable high-resolution data set for sickness model identification.

### Kinematic and physiological variables

For the analysis of the physiological measurements, the present study employed a methodology whereby the sample population was not arbitrarily segmented in sick and not-sick groups, as has often been done in the past. Motion sickness occupies a spectrum of severity and clear cut-offs may introduce undesired effects. Linear mixed models with random intercepts were used instead to separate trends in time from effects of motion sickness and to quantify these contributions to MISC.

For the tonic GSR, there is a significant increase with both time and sickness. This is very much expected and likely due to increasing sweating due to the stabilizing action of the muscles when subject to the 0.4 g lateral accelerations. For the phasic GSR, there is a significant increase with respect to sickness, but not with time. This is also expected, as the phasic component is more indicative of sympathetic activation (Benedek and Kaernbach 2010a). There is a larger effect of sickness on the phasic component, i.e., 90% of the baseline, whereas the increase of the tonic GSR is 42.5% of the baseline.

For the heart rate and the LF/HF ratio, the results are found to be more mixed. MISC seems to elevate the heart rate, but this effect is small compared to the general decrease in heart rate due to relaxation from the high state of arousal at the start of the experiment. Thus, the strength of the link between heart rate and motion sickness level likely depends on the nature of the experimental stimulus. Under the extreme scenario tested here, time effects are likely to be larger than in most laboratory experiments. In the present study, we observed a small but significant decrease of the LF/HF ratio, which constituted 18.52% of the baseline LF/HF ratio. This measure is traditionally taken as an indicator of sympatho-vagal balance, whereby an increase in the ratio is indicative of increased sympathetic activity. As motion sickness activates the sympathetic nervous system, an increased LF/HF ratio is expected. This activation is evident in the GSR responses. Recently, however, it has come to attention that LF/HF ratio is not a simple linear measure of sympatho-vagal balance, but is instead a much more complex and non-linear metric. For instance, it is now known that LF power of heart rate variability is also modulated by the parasympathetic nervous system, and vice versa for the HF power of heart rate variability (Billman 2013). Therefore, LF/HF ratio is a complex measure of sympathetic and parasympathetic activity which on its own, as seen in our results, is not likely to be an effective indicator of motion sickness.

In our experiment, we found head roll to increase with exposure to sickening motions. Our regression model showed this to relate significantly only to exposure time. Exposure time relates to fatigue and/or the willingness to go with the motions, rather than opposing them. The nonsignificant relation with sickness does not support the postural instability theory of motion sickness (Stoffregen and Smart 1998). However, this may owe to the noisy nature of the experimental data which were collected from sitting participants subject to high accelerations, as well as the filtering process required to obtain the results. Regardless of the outcome, we could have made no statements on whether postural instability preceded sickness, or rather results from sickness. The latter seems more probable in terms of decreased control due to sensory conflict (Bos 2011). There is evidence that roll/pitch of the head when combined with linear translational accelerations interact in a non-linear manner to increase sickness to levels higher than would be possible if they were experienced in isolation (Wertheim et al. 1998). On the other hand, Golding et al. (2003) observed that the effect of head roll on sickness depended on whether it was active or passive. Here, it was found that active alignment of the head with the direction of the gravito-inertial acceleration would protect against motion sickness whereas active movements against would increase symptoms of motion sickness. For passive motions (created by actuation of the passenger seat), on the other hand, the opposite was the case. The rotations experienced in the current study cannot be characterized as active or passive. Therefore, whether head roll acts as positive feedback mechanism to increase sickness levels cannot be stated.

Of all considered metrics, the phasic GSR correlates most strongly with MISC, increasing 90% above its baseline value at MISC 7. However, the increase only becomes noticeable when averaging over the entire population and after prolonged exposure. It remains to be proven how well this measure can be used as individual (real time) classifier of motion sickness, to complement or even replace subjective assessments such as the MISC.

### Implications

We confirm that having external vision strongly reduces motion sickness so vehicle manufacturers and also passengers should design and act to maximize world referenced visual information. This can include novel technologies that might increase anticipatory cues, some of which were discussed in "Internal–external vision".

Knowing the dynamics and parameters of hypersensitivity may enable route planning that accounts for motion sickness. For instance, sections of road that provide large sickening stimulus, i.e., traffic or mountain road may be scheduled such as to provide adequate reduction in sensitivity in between exposures.

More importantly, by showing that motion sickness is regular and repeatable within individual participants we now have a greater basis for using system-theoretical, model-based approaches for understanding motion sickness.

Lastly, validating the Oman model as an approximate model for sickness dynamics, opens the door for developing robust platform controllers that may allow for the control of sickness levels in individual participants to a desired reference level.

## Conclusion

From experiments where we measure the development of motion sickness during a highly sickening drive, we find a significant increase in mean MISC, from 2.3 (at the end of exposure) in the external vision condition to 4.2 in the internal vision condition. We believe this is largely due to a reduction of anticipatory cues, rather than removal of visual translational and yaw rotation cues.

We show that individuals exhibit a wide variation in the dynamics of sickness development over time, whose key individual characteristics are masked by group averaged metrics, as often used in previous publications. We, for the first time, attempt to fit Oman’s model of nausea to time-domain data of individual participants. We see that these fits are able to accurately model the full course of individual’s sickness development for our experimental data, including the rest and hypersensitivity phases.

Using this model, we show that motion sickness is a repeatable phenomenon with individual motion sickness responses showing a high degree of repeatability over consecutive sessions. GSR responses show significant effects of exposure time and of sickness level. These effects are highly significant at the group level with exposure for up to 30 min or until a MISC of 7 is reached, but show substantial scatter. Due to this natural spread, such objective measures are unlikely to replace subjective sickness measures (e.g., MISC) for real-time motion sickness classification.
